# Role of CaMKII in diabetes induced vascular injury and its interaction with anti-diabetes therapy

**DOI:** 10.1007/s11154-023-09855-9

**Published:** 2023-12-08

**Authors:** Stephanie Chacar, Abdulhamid Abdi, Khalifa Almansoori, Jawaher Alshamsi, Cynthia Al Hageh, Pierre Zalloua, Ali A. Khraibi, Stephen G. Holt, Moni Nader

**Affiliations:** 1 Department of Physiology and Immunology, College of Medicine and Health Sciences, Khalifa University of Science and Technology, Abu Dhabi, United Arab Emirates; 2 Department of Molecular Biology and Genetics, College of Medicine and Health Sciences, Khalifa University of Science and Technology, Abu Dhabi, United Arab Emirates; 3 Center for Biotechnology, Khalifa University of Science and Technology, 127788 Abu Dhabi, United Arab Emirates; 4 SEHA Kidney Care, SEHA, Abu Dhabi, UAE

**Keywords:** CaMKII, Diabetes, Vasculature injury, Vascular smooth muscle cells, Endothelial cells

## Abstract

Diabetes mellitus is a metabolic disorder denoted by chronic hyperglycemia that drives maladaptive structural changes and functional damage to the vasculature. Attenuation of this pathological remodeling of blood vessels remains an unmet target owing to paucity of information on the metabolic signatures of this process. Ca^2+^/calmodulin-dependent kinase II (CaMKII) is expressed in the vasculature and is implicated in the control of blood vessels homeostasis. Recently, CaMKII has attracted a special attention in view of its chronic upregulated activity in diabetic tissues, yet its role in the diabetic vasculature remains under investigation.

This review highlights the physiological and pathological actions of CaMKII in the diabetic vasculature, with focus on the control of the dialogue between endothelial (EC) and vascular smooth muscle cells (VSMC). Activation of CaMKII enhances EC and VSMC proliferation and migration, and increases the production of extracellular matrix which leads to maladaptive remodeling of vessels. This is manifested by activation of genes/proteins implicated in the control of the cell cycle, cytoskeleton organization, proliferation, migration, and inflammation. Endothelial dysfunction is paralleled by impaired nitric oxide signaling, which is also influenced by CaMKII signaling (activation/oxidation). The efficiency of CaMKII inhibitors is currently being tested in animal models, with a focus on the genetic pathways involved in the regulation of CaMKII expression (microRNAs and single nucleotide polymorphisms). Interestingly, studies highlight an interaction between the anti-diabetic drugs and CaMKII expression/activity which requires further investigation. Together, the studies reviewed herein may guide pharmacological approaches to improve health-related outcomes in patients with diabetes.

## Vascular smooth muscle cells and endothelial cells: the major constituents of the arterial wall

The structure of the normal artery consists of three main layers: the intima, the media, and the adventitia [1]. The media is mainly composed of vascular smooth muscle cells (VSMCs), whilst the intima constitutes the innermost monolayer of endothelial cells (ECs). The occasional existence of VSMCs in the intima of children has been reported, while an infiltration of VSMCs into the intima may occur with aging or following vessel injury [2–5]. The adventitia harbors a mixture of fibroblasts and VSMCs along with components of the extracellular matrix (e.g. collagen and proteoglycans) [6].

In blood vessels, VSMCs play a major role in vessel contraction and the control of its diameter, and thus the distribution of blood flow. VSMCs usually proliferate at a low rate and possess poor synthetic activity [7], but under specific conditions proliferation and phenotypic changes contribute to the development of atherosclerosis, arteriosclerosis, and hypertension [8]. In response to various factors, VSMCs can reversibly switch from a “contractile” to a “synthetic” phenotype, and proliferate [9]. For example, the platelet-derived growth factor (PDGF), which is released during vessel injury, is pivotal for the proliferation of VSMCs [10, 11].

ECs are restricted to the intima, and they are structurally and functionally coupled to the VSMCs and they regulate vascular function and structure. The interaction between ECs and VSMCs starts early as ECs signal to VSMCs to cause differentiation at embryonic stages by inducing the expression of smooth muscle cell specific markers (SM-Myosin, SM22alpha, and calponin) [12–14]. ECs release vasoactive substances that control the relaxation, contraction, and growth of VSMCs, especially nitric oxide (NO), prostaglandins, and endothelium-derived hyperpolarizing factor to specifically induce vasodilation [3, 15, 16]. This crosstalk between ECs and VSMCs appears impaired during remodeling of blood vessels. Remodeling may be manifested by either pathological lesions, changes (increase/decrease) in the thickness of the media or the lumen of the vessels, functionally reflected by changes in blood flow and blood pressure dynamics [17, 18].

The maladaptive changes in the vascular wall have been the major focus for therapeutic interventions in various conditions i.e. hypertension, stenosis/restenosis, inflammation and diabetes [17–21]. In fact, whilst various diabetes-related factors could contribute to vasculopathies, hyperglycemia remains a major cause of the pathological remodeling of the micro- and macro-vasculature [22], thus contributing to vascular pathology in diabetic subjects [14]. Endothelial impairment contributes to the development of atherosclerosis and vascular disease [23, 24], with coronary and peripheral arterial disease highly prevalent in diabetic patients [25–27]. Chronic hyperglycemia in diabetes results in oxidative stress and activation of protein kinase C (PKC) and polyol pathways [23, 28, 29] that might contribute to proliferation of vessels and altered signal transduction [30]. Further, hyperglycemia encourages attachment of inflammatory cells to the endothelium of the vasculature [23, 31].

There are several signaling networks that are involved in the pathological remodeling of blood vessels under hyperglycemic conditions, one of these that appears to be important is the Calcium/Calmodulin-dependent protein kinase II (CaMKII) pathway. This pathway is also implicated in diabetic cardiomyopathy [32–35]. Thus CaMKII has recently emerged as a possible therapeutic target in various conditions (reviewed in [36]). In the subsequent sections, we will examine the role of CaMKII in diabetes-induced remodeling of the EC-VSMC interaction and blood vessel homeostasis, with a focus on anti-diabetic therapies and their effect on CaMKII signaling.

## Characterization of the four CaMKII isoforms and their function

CaMKII, a calcium (Ca^2+^)-sensing enzyme, is a serine-threonine kinase with a dodecameric structure [37]. Each monomer consists of three domains, an N- terminal catalytic domain that incorporates the kinase activity, an autoregulatory domain that mediates the function of the catalytic domain, and a C-terminal end that drives multimeric assembly [38]. It is a member of a large family of diverse CaM kinases including CaMKI, II and IV. CaMKII is distinguished by its four isoforms α, β, γ, and δ that are encoded by separate genes [39]. CaMKII α and β are predominantly expressed in the brain and also in ECs, whilst CaMKII δ and γ are found in a broader range of tissues, particularly in the heart [40, 41]. The gene coding for the δ variant is the main isoform expressed in cultured VSMCs with δ2 being the major CaMKII splice variant [42], but differentiated VSMCs express both CaMKII γ and δ [43].

An increase in intracellular Ca^2+^ triggers the activation of all CaMKII isoforms and coordinates ion channel and proteins involved in Ca^2+^ homeostasis [44, 45]. In neurons, CaMKII is critically involved in complex cognitive and behavioral responses, including synaptic plasticity, learning, and long-term memory [46, 47]. At the cardiac level, CaMKII is crucial for excitation–contraction coupling and excitation–transcription coupling that occurs in cardiomyocytes. Not only is CaMKII critical for normal function of the myocardium, but it also plays a central role in signaling pathways mediating pathological remodeling of the heart [48]. For example, oxidized CaMKII enhances proinflammatory cytokines by upregulating nuclear factor- _k_B (NF_k_B) expression [49].

Whilst most studies have been carried out on neuronal cells and cardiomyocytes, some evidence suggests that CaMKII also plays an essential role in the regulation of glycogenolysis/gluconeogenesis [50], and the immune system [51].

## Implication of CaMKII in vascular physiology

A growing body of evidence suggests that CaMKII exerts a central effect on the function of vascular cell types, specifically the ECs and VSMCs [45, 52]. It has been implicated in the regulation of EC proliferation, migration and inflammation [53]. CaMKII may also be important in the regulation of endothelial nitric oxide synthase (eNOS) and inducible nitric oxide synthase (iNOS) enzymes [54]. When these enzymes are activated, they cause nitric oxide (NO) release locally which induces relaxation in VSMCs and thus vasodilatation [55]. Following stimulation by receptor-dependent and -independent agonists, there is an increase in the intracellular cytosolic concentrations of free Ca^2+^. When Ca^2+^ levels rise, Ca^2+^/calmodulin complexes bind to CaMKII, triggering its activation, which enhances NO production in ECs. CaMKII has been shown to increase eNOS activity by direct phosphorylation of the enzyme, which enhances its catalytic activity and results in increased production of NO. The phosphorylation promotes eNOS coupling with its cofactor, tetrahydrobiopterin (BH4). BH4 is a critical cofactor for the production of NO, it preserves eNOS dimerization and improves endothelial function [56, 57]. This activation has been shown to play a critical role in regulating vascular tone and blood pressure [54]. CaMKII has also been shown to modulate iNOS expression and activity. Inhibiting CaMKII activity causes an accumulation of a CaMKII/iNOS complex in an aggresome-like structure with consequent decrease in iNOS activity [58].

### CaMKII in VSMCs

CaMKII is abundantly expressed in VSMCs [38] suggesting a role in vasoconstriction [45]. A study by Humphries et al., was the first to explore the role of CaMKII in initiating cellular activity linked to vasodilatation. It revealed that exchange protein directly activated by cAMP (Epac)-induced spontaneous transient outward currents (STOCs) activity in contractile vascular smooth muscle occurs via the activation of CaMKII and thus induces relaxation in VSMC by increasing the sensitive release channels of peripheral sarcoplasmic reticulum ryanodine receptor (RyR) [59].

CaMKII has been shown to be involved in the regulation of L-type Ca^2+^ channels, potassium channels [60], as well as VSMCs migration [61] and proliferation [62]. Numerous factors promote VSMCs migration including PDGF stimulation, reactive oxygen species (ROS), and matrix metalloproteinase-9 (MMP-9) [32, 63–65]. PDGF and other growth factors cause VSMC migration that is associated with a rapid activation of CaMKII [64]. A cytoplasmic increase in Ca^2+^ causes enhanced CaMKII activity during VSMCs migration [66]. ROS-activated CaMKII stimulates VSMCs migration which is evident following vascular damage [67]. CaMKII is one of the key downstream targets of ROS signaling. ROS oxidize and activate CaMKII, leading to its autophosphorylation at threonine 287 and its sustained activation. Activated CaMKII subsequently catalyzes phosphorylation of proteins and transcription factors involved in VSMCs migration. CaMKII is also activated by methionine 281/282 oxidation (ox-CaMKII), which maintain CaMKII in an active state [68]. Carriers of a mutant CaMKII (by knock-in replacement of methionine 281 & 282 with valine) exhibited reduced oxidation-dependent activation of CaMKII and decreased VSMCs migration [69, 70].

Under normal circumstances, VSMCs remain in a non-proliferative state (G0), however, they enter the cell cycle when provoked by injury. Several mediators/regulators of the cell cycle appear to be influenced by CaMKII and include PDGF, cyclin-dependent kinase 2 (Cdk2), and cyclin E. CaMKII activation promotes production of Cdk2 and cyclin E, which drives transition from G1 to S phase. The cell cycle down regulator p21 reduces Cdk2 and cyclin E activity and in CaMKIIδ^−/−^ mice with carotid artery injury, p21 is overexpressed compared with control mice, which exhibits marked inhibition of VSMCs proliferation [62, 71].

The cAMP responsive element binding protein (CREB) downregulates VSMC growth; CREB is itself inhibited by CaMKIIδ2 (through Ser142 phosphorylation) enhancing VSMC growth [72]. Passaged primary cultures of de-differentiated VSMCs predominantly express CaMKIIδ2 that promote transition from G1 to G2/M phase of the cell cycle [43].

**Fig. 1:**
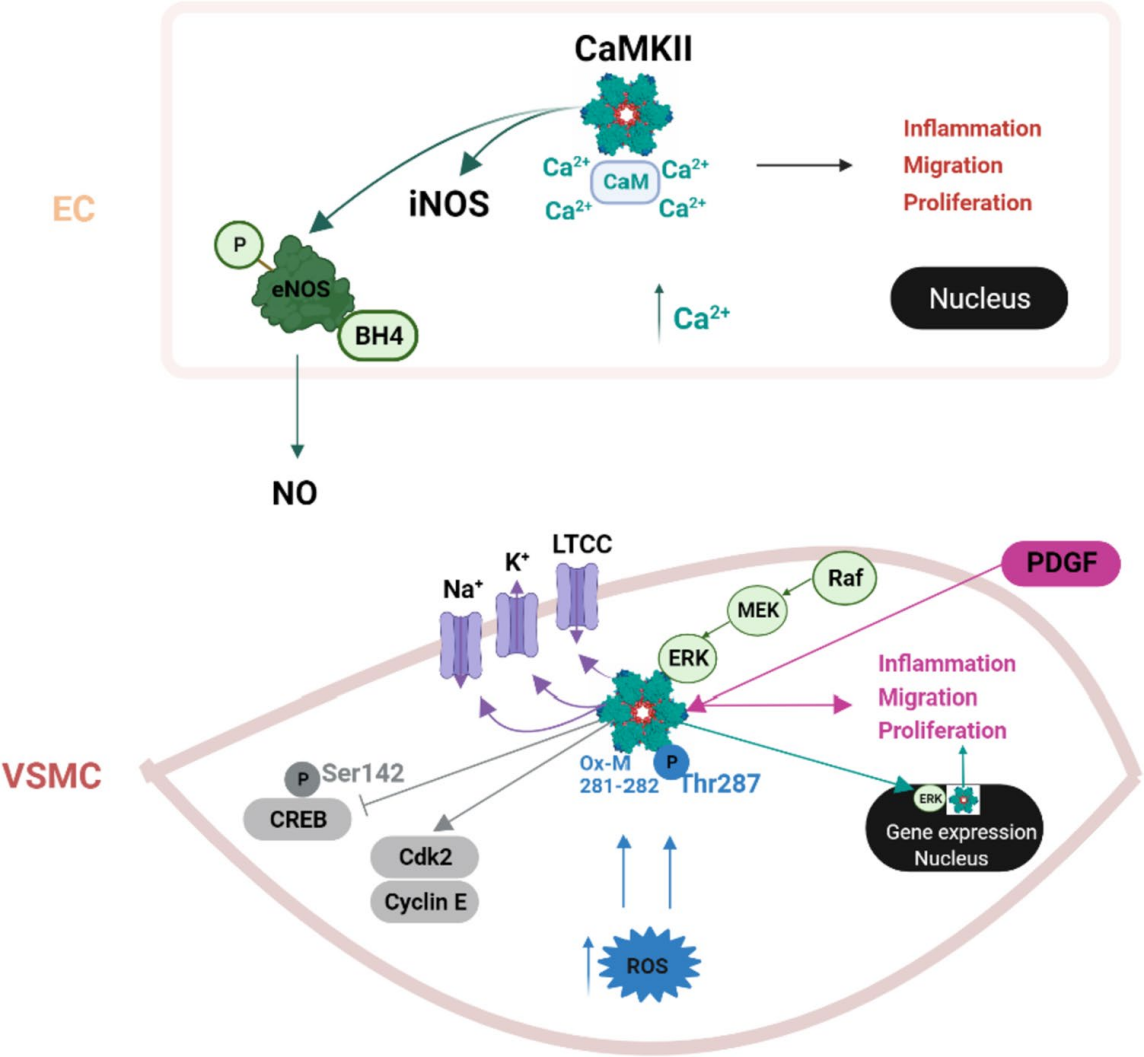
Control and function of CaMKII in ECs and VSMCs. Schematic drawing summarizing the major regulators of CaMKII function in ECs and VSMCs. The lines/effectors of same color depict a signaling cascade and the role of CaMK on the effectors in EC and VSMC. Abbreviations: EC: endothelial cell; VSMC: vascular smooth muscle cell; CaMKII: Ca^2+^/calmodulin-dependent kinase II; iNOS: inducible nitric oxide synthase; eNOS: endothelial nitric oxide synthase; BH4: tetrahydrobiopterin; LTCC: L-type calcium channel; Na^+^: Sodium; K^+^: potassium; PDGF: platelet-derived growth factor; ERK: extracellular-signal-regulated kinase; CREB: cAMP responsive element binding protein; Cdk2: cyclin-dependent kinase 2; ROS: reactive oxygen species; Ox-M: Ox-Methionine. Figure created with Biorender.com

Another important role of CaMKII in VSMCs proliferation is exerted by the Raf/MEK/ERK pathway. Activated CaMKII forms a protein complex with the extracellular-signal-regulated kinase (ERK), but does not directly phosphorylate it. CaMKII rather activates Raf1, which indirectly causes ERK phosphorylation (via MEK) and this complex translocates to the nucleus, promoting proliferation [73]. Together, these findings suggest an important role for CaMKII in VSMCs proliferation *in vitro* and *in vivo*. Figure 1 summarizes the dynamics of CaMKII in ECs and VSMCs.

## Role of CaMKII in vascular injury in diabetes mellitus

### CaMKII-dependent regulation of ECs structure and function in diabetic conditions

Although CaMKII is primarily involved in the pathogenesis of direct vascular damage e.g. that caused by pressure injury (hypertension), it is not yet clear whether it is as involved when such damage occurs secondary to other insults (e.g. diabetes) [74].

In diabetic environment vaso-responsiveness is significantly impaired. For example, *in vitro*, both the vasodilation to NO and the vasoconstriction to endothelin-1 are significantly attenuated [75–77]. These are further impacted by functional deterioration of the peripheral sympathetic nervous system [75, 78], increased activity of PKC, expression of pro-inflammatory transcription factor nuclear factor kappa B (NF-κΒ), and generation of oxygen-derived free radicals [75, 79, 80].

In contrast, induced NO release from lipopolysaccharide which upregulates iNOS, is markedly increased in diabetic rats compared to their control counterparts [35]. This increase is followed by an increase in intracellular Ca^2+^ and activation of CaMKII via phosphorylation. A role for CaMKII in endothelial injury has been shown using chronic CaMKII inhibition (with KN-93) which halted the progression of endothelial dysfunction in diabetic rats [81]. This could be due to the effect of CaMKII on the subcellular localization of filamin, an actin-binding protein implicated in the rearrangement of the cell cytoskeleton and the control of cell size and shape, as well as the regulation of gene expression [82]. CaMKII promotes the formation of tight junctions (TJ) in ECs in an Adenosine Monophosphate-Activated Protein Kinase (AMPK) and claudin-1 dependent manner [83]. The inhibition of CaMKII using KN93 causes a spontaneous polymerization of claudin-1, which promotes the development of TJs [83]. Interestingly, CaMKII also influences thrombin/fibronectin-induced EC migration, which points to CaMKII as a target for pathological remodeling of blood vessels [84]. The reorganization of these structural proteins could be impaired in diabetic conditions. These findings suggest that CaMKII may be a driver of endothelial dysfunction in hyperglycemic conditions, independent of NO signaling. It is worth noting that CaMKII inhibition enhances the contraction and relaxation of myocardial tissue in diabetic rats, which suggests that it may have an action on cardiac myocytes as well [85].

Based on these findings, potential therapeutic strategies revolving around the inhibition of CaMKII may be developed for the treatment of diabetes-associated vascular damage.

### CaMKII-dependent regulation of VSMCs in diabetic conditions

In contrast to ECs, VSMCs are organized in multiple layers across the wall of blood vessels and, as indicated earlier, their remodeling in diabetes translates into many pathological processes including hypertension, atherosclerosis, and plaque rupture. The interplay of CaMKII with ROS in diabetic VSMCs may contribute to vasculopathies [86]. It has been shown that hydrogen peroxide (H_2_O_2_) and/or glucose induced phosphorylation of ERK1/2 in VSMCs is under the control of CaMKII, and this promotes growth, proliferation, and hypertrophy of VSMCs [87]. Additionally, either the inhibition, or the silencing of CaMKII (in H_2_O_2_ stimulated cells) attenuates phosphorylation of the insulin-like growth factor-1 receptor (IGF-1R), suggesting a role for CaMK in insulin metabolism and implies a diabetogenic role for this enzyme [87, 88]. On the other hand, the pharmacological challenge of VSMCs (from diabetic rats) with lipopolysaccharide showed increased expression of iNOS, activated CaMKII, but a weaker interaction between iNOS and CaMKIIδ2 compared with non-diabetic animals [35]. These studies suggest that CaMKII activity and its interaction with iNOS are altered in VSMCs under hyperglycemic conditions. In ECs, CaMKIIδ contributes to the production of ROS via phosphorylation of NADPH oxidase [63]. Following vascular insult, the expression of CaMKIIδ2 is promoted [43, 89], resulting in the activation of the MAPK pathway [43, 89, 90], increased proliferation and migration, which ultimately manifests as neo-intimal hyperplasia [43, 89]. Thus, these studies suggest that the maladaptive remodeling of blood vessels in hyperglycemic conditions may result from an aberrant activity of isoforms of CaMKII. Other studies have shown that CaMKIIδ involvement in diabetic injuries is tissue- and cell-type specific: this is evident in CaMKIIδ-deficient mice (crossed with leptin receptor-mutant mice) that do not develop hyperglycemia, but show improved glucose transport into skeletal muscle and reduced production of hepatic glucose. However, these mice exhibited diabetic nephropathy without diabetic retinopathy, most probably due to differences in glucose metabolism between the kidneys and the retina [91]. The authors proposed that CaMKIIδ may phosphorylate the insulin receptor to block its activity and inhibit the expression of GLUT4, as highlighted by other studies [92, 93]. While pharmacological inhibition (KN93) of CaMKII attenuates the activity of CaMKII in diabetic vessels, there is urgency to define the molecular cues behind the CaMKII isoforms-induced diabetic vasculopathy, particularly CaMKIIγ and δ that are predominantly expressed in VSMCs, to better guide therapeutic interventions focused on either ECs, or VSMCs, or probably both.

## Impact of CaMKII on macrophages and progenitor cells: possible therapeutic targets in diabetes?

The reduced ambient NO levels in patients with diabetes may contribute to enhanced expression of leukocyte adhesion molecules, chemokines and cytokines. These actions promote monocyte migration into the intima and the formation of foam cells from macrophage [75]. The accumulation of macrophages in the vascular wall causes plaque instability and rupture by reducing VSMCs proliferation. Within the unstable plaque, CaMKII expression is increased in macrophages and actively regulates chemokine production [94]. Knockout models of CaMKII in cardiomyocytes showed a reduction in chemokines which implies that this may also occur in the vasculature [95].

Endothelial progenitor cells (EPCs) are also implicated in the repair process of the damaged blood vessel. EPCs are damaged in type 2 diabetes (T2D), with few studies suggesting that EPC dysfunction precedes the onset of the disease itself [96]. This defect in EPCs leads to a lower recruitment of progenitor cells to the site of injury, which may manifest as nephropathy [97], retinopathy [98], and cardiomyopathy [99] in diabetic patients. Taking retinopathy as an example, this disease becomes clinically noticeable when the microenvironment in the eye is altered by proangiogenic factors, thus favoring neovascularization. In contrast to other areas in the body, this neovascularization promotes endothelial progenitor cell recruitment, further exacerbating the degree of retinopathy [98]. In this context, CaMKII contributes to pathological retinal angiogenesis, and appears to be a key signaling step in angiogenic activity in human retinal ECs. In fact, the signaling of several angiogenic molecules including fibroblast growth factor, hepatocyte growth factor, insulin-like growth factor-1, and the vascular endothelial growth factor, converge towards CaMKII in the retina. Whilst these factors induce cell proliferation and migration, pharmacological inhibition of CaMKII (KN93) abolished these effects [100]. These data crystallize CaMKII as a novel therapeutic target for diabetes-induced retinopathies.

CaMKII is also involved in the proliferation of hematopoietic stem and progenitor cells in response to bone marrow injury [101]. Here, CaMKII knockout mice exhibited faster regeneration of peripheral blood and bone marrow injury, and they were more resilient to radiation-induced insults compared with CaMKII intact mice. This crystallized CaMKII as a negative regulator of cell proliferation and highlighted a possible role for CaMKII inhibition in enhancing cell expansion and recovery. However, this is difficult to reconcile with other findings where CaMKII was found to be proliferative and stimulated migration in retinal ECs, suggesting tissue specificity in CaMKII actions [98].

The role of the oxidation of CaMKIIδ in arteriogenesis and the infiltration of macrophages in the perivascular space has also been studied [65]. Outward remodeling of arteries appears to be controlled by the oxidation of CaMKIIδ and the secretion of MMP-9 by macrophage. Studies have identified CaMKIIδ as a mediator of ROS-signaling in the vasculature, and highlighted a role for CaMKII in diabetic vasculopathy due to the activation of ROS in this setting. Further, endogenous CaMKIIγ serves as a functional brake on CaMKIIδ-mediated signaling, which otherwise promotes VSMCs proliferation and vascular wall remodeling, hinting at different repair functions amongst the various CaMKII isoforms [102]. In addition, CaMKIV, which is mainly expressed in the brain and hematopoietic stem cells (HSC), may be essential for cell survival [103]. Taken together, these studies underscore a role for CaMKII in the repair and maintenance of the injured blood vessels.

## CaMKII miRNA targets in the vasculature and its genetic polymorphism in diabetes

MicroRNAs (miRNAs) are a class of small non-coding RNAs that modulate post-transcriptional protein expression and have been suggested to have a key regulatory role in diverse biologic processes [104]. They emerged as regulators of several pathophysiological conditions including vasculoproliferative diseases. The expression of CaMKIIδ in VSMCs may be regulated by miR-30 family members (miR-30), miR-143, and miR-145 following vascular injury [105]. It appears that miR-30 inhibits CaMKIIδ-dependent VSMCs function and neointimal VSMCs hyperplasia induced by vascular injury. Interestingly, the miR-143/145 cluster overexpressed in VSMCs obtained from rat aorta with a contractile phenotype, also inhibit CaMKIIδ protein expression [105, 106]. Both miR-143 and miR-145 also regulate the insulin signaling pathway and glucose uptake in VSMCs. Deletion of the miR-143/145 cluster results in potentiated insulin signaling and insulin-induced glucose uptake [106]. Thus miR-143/145 may be endogenous inhibitors of CaMKII, but enhance insulin-induced glucose uptake, providing perhaps a novel therapeutic target for diabetes. Several single nucleotide polymorphisms (SNPs) have been identified in the genes encoding CAMK2 isoforms. The SNPs have been found to be associated with a range of phenotypes, including T2D, Parkinson's disease, Alzheimer's disease, atrial fibrillation, chronic bronchitis, human height, and liver-related alkaline phosphatase disease. A summary of the relationship between the phenotypes and the associated SNPs in CAMK2 isoforms is provided in Table 1. These associations suggest that genetic variation in CAMK2 isoforms may contribute to the development or modulation of the clinical manifestations of a number of the above mentioned disorders.

**Table 1:** Single Nucleotide Polymorphisms (SNPs) in CAMK2 Isoforms

**Gene**	**SNP**	**Trait**	**Class**	**References**
*CAMK2G*	rs2633310	T2D	Disease	[107]
*CAMK2G*	rs2633311	T2D	Disease	[108]
*CAMK2G*	rs2675662	T2D and coronary artery disease	Disease	[109]
*CAMK2D*	rs11768656 rs7978469 rs79582698 rs62459110 rs78454627	T2D	Disease	[110]
*CAMK2B*	rs35452727	T2D	Disease	[111]
*CAMK2B*	rs878521	T2D	Disease	[112]
*CAMK2B*	rs2003563	Reduced Alkaline phosphatase (liver-related disease)	Biomarker	[111]
*CAMK2G*	rs60820984	Reduced Lung function, chronic obstructive pulmonary disease (COPD)	Disease	[113]
*CAMK2G*	rs7098573	Reduced Lung function, chronic obstructive pulmonary disease (COPD)	Disease	[113]
*CAMK2A*	rs2053053	Persistent and repetitive antisocial behaviour (conduct disorder)	Disease	[114]
*CAMK2A*	rs3756577	Alzheimer and mild cognitive impairment	Disease	[115]
*CAMK2D*	rs13117519	Parkinson's disease	Disease	[116]
*CAMK2D*	rs17483653	Parkinson's disease	Disease	[117]
*CAMK2D*	rs78738012	Parkinson's disease	Disease	[118]
*CAMK2D*	rs55754224	Atrial fibrillation	Disease	[119]
*CAMK2D*	rs6829664	Atrial fibrillation	Disease	[120]
*CAMK2D*	rs80195545	Chronic bronchitis	Disease	[111]
*CAMK2A*	rs17111053	Heritable Human height difference	Anthropometric	[121]
*CAMK2B*	rs7797038	Heritable Human height difference	Anthropometric	[121]
*CAMK2G*	rs2242258	Heritable Human height difference	Anthropometric	[121]
*CAMK2D*	rs1859229	Heritable Human height difference	Anthropometric	[121]
*CAMK2D*	rs7669672	Heritable Human height difference	Anthropometric	[122]

## Pharmacologic and genetic control of CaMKII

The use of pharmacologic inhibitors of CaMKII activity to date has been mainly limited to empirical research with little or no translation to interventions, at least in humans. There are different approaches to suppress the signaling of CaMKII and these include direct pharmacological inhibitors using small molecules (e.g. KN-93, AS105 and GS-680), peptides, and RNA interference (RNAi).

KN-93, one of the first CaMKII inhibitors, is an allosteric inhibitor preferentially binding to CaMKII in the inactive state [32, 123]. This mechanism of inhibition is thought to be less efficient at inhibiting non-active CaMKII, including autonomously activated CaMKII (from Thr287 auto-phosphorylation). Unfortunately KN-93 is poorly selective, with a half maximum inhibitory concentration (IC50) in the 1 to 4 µM range [124]. Nevertheless, it has been useful as a research tool, and it has emerged lately as a focus for pharmacological inhibition of CaMKII in clinical settings [125]. A novel CaMKII inhibitor, AS105, was developed by Allosteros Therapeutics as an ATP-competitive pyrimidine-based molecule. AS105 displayed a remarkable *in vitro* IC50 of 8 nM and effectively mitigated Ca^2+^ dysregulation in adult cardiomyocytes from CaMKIIδC-overexpressing mice. Importantly, AS105 inhibition of CaMKII had no adverse effects on baseline Ca^2+^ handling, consistent with results from CaMKII knockout mice [126]. These findings surface the potential of AS105 as a specific and effective CaMKII inhibitor, holding a promising therapeutic applications in the treatment of cardiac conditions.

GS-680 is another novel selective and ATP-competitive CaMKII inhibitor that has been developed by Gilead Sciences, with an IC of 2.3 nM against CaMKIIδ. Biopsies from patients undergoing surgeries revealed that GS-680 demonstrated effectiveness in preventing CaMKII-dependent proarrhythmic activity and in reducing sarcoplasmic reticulum Ca^2+^ leak in human atrial trabeculae [127].

Peptide inhibitors have been developed and studied as potential inhibitors of CaMKII. Autocamtide-2-related inhibitory peptide (AIP) [128] and autocamtide-3 derived inhibitory peptide (AC3-I) [129] are designed based on the structure/sequence of the auto-regulatory domain of CaMKII. These inhibitors mimic the pseudosubstrate domain, with a key mutation at T287 to a phosphorylation-resistant alanine, effectively sequestering the catalytic domain. In contrast with the complete CaMKII structure, these peptides lack the CaM binding domain, enabling them to remain bound even in the presence of Ca^2+^/CaM [130]. Consequently, these inhibitors have been employed both pharmacologically and genetically to effectively impede CaMKII's pathological signaling. CaMKIIN including CaMKIINα and CaMKIIβ were subsequently identified. These peptides selectively inhibit CaMKII activity with an IC50 of 50 nM [131]. CaMKIIN molecules exhibit a higher degree of selectivity by directly binding with the kinase domain in the active conformation. Peptide inhibitors are genetically encoded, they can be specifically delivered to selective regions by using locally administered viral vectors and guided to specific intracellular locations with suitable targeting sequences [132].

On the other hand, targeted gene silencing is a more selective tool to suppress CaMKII expression and activity. Here single-stranded antisense oligonucleotides, double-stranded siRNAs, or miRNAs [133] are used to knock-down CaMKII expression. Although theoretically downregulating CaMKII with RNA based therapy looks promising, current agents are delivered systemically either by intravenous or intramuscular injections, which adds to the complexity of selectively targeting CaMKII in specific tissues. Recently, CRISPR-Cas9 targeting CaMKIIδ in cardiomyocytes from human induced pluripotent stem cell effectively suppressed the oxidation of methionine on CaMKII, thus rendering these cells more tolerant to ischemia-reperfusion injuries [134]. Moreover, ablating the autophosphorylation site of CaMKIIδ using CRISPR-Cas9 in mice protected their hearts from transaortic constriction induced heart failure [135]. Together, these studies offer hope for direct targeting of CaMKII domains to better control its dynamics in diabetes settings. To our advantage, this gene editing approach is permanent and may provide a unique therapeutic tool to control CaMKII activity in patients with diabetes [136]. Together, these studies offer hope for direct targeting of CaMKII domains to better control its dynamics in diabetes settings [137].

## Intersection of the anti-diabetes therapy and CaMKII signaling pathways

Upregulation and constitutive over-activity of CaMKII are features of several pathologies in diabetes mellitus including heart failure and possibly chronic kidney disease (CKD). It is therefore helpful to evaluate whether the anti-diabetes therapy can modulate CaMKII signaling.

It is well known that excess calorie intake and a sedentary lifestyle are associated with the development of T2D. The literature contains a few studies linking lifestyle interventions to the expression/activity of CaMKII. One study examined the impact of exercise and endurance training on angiogenesis-related genes in cardiac tissue of diabetic rats. It was found that six weeks of moderate-intensity exercise training reduced the expression of CaMKII and allowed more effective control of glucose homeostasis by improving glucose uptake in adipose and muscle tissue [138]. Another study reported that a chronically high salt intake induced an increase in intracellular ROS and high CaMKII activity which led to impaired endothelial function [139]. Studies in obese individuals show that adipocytes have higher intracellular mitochondrial Ca^2+^ levels and reduced glucose uptake [140]. Inhibition or deletion of CaMKII in the liver of obese mice protects against insulin resistance and this is similar in adipose tissue [141, 142]. Interestingly, in a study of diabetic mice (leptin receptor mutation) crossed with CaMKII knockout mice, there was no development of hyperglycemia, but normal glucose uptake into muscle, reduced hepatic glucose synthesis, and the absence of retinopathy. However, despite normoglycemia, these mice still developed diabetic nephropathy, suggesting that hyperglycemia *per se* is a symptom, and not a direct cause, of diabetic nephropathy. However, CaMKII may be directly involved in nephropathy progression as it has been shown that CaMKII upregulation contributes to renal fibrosis [143] and enhanced matrix production in CKD [144].

Many drugs may directly or indirectly interfere with or affect CaMKII signaling. Insulin stimulates growth and proliferation of many cells involving CaMKII signaling pathway and CaMKII signaling may be further involved in signals downregulation after insulin stimulation [93]. CaMKII appears to modify insulin-induced ERK1/2 activation and cell proliferation, and after stimulation, CaMKII mediates the down-regulation of glucose uptake. Thus, CaMKII may play a central role in long term insulin response and modulation of this pathway may enhance insulin action. Somewhat counterintuitively, the biguanide metformin, has been shown to enhance CaMKII dependent protein synthesis in mouse liver [145]. Nevertheless, there is paucity of data on the effects of biguanides on CaMKII activation. Biguanides and sulphonylureas are widely used in the treatment of T2D, and whilst there is little direct evidence on the effect of these drugs on CaMKII pathways, there is some tangential evidence that they may change the CaMKII signaling pathway. The sulphonylurea, glyburide, activates CaMKII and enhances anti-inflammatory responses which improves wound healing in a high fat diet obese mouse model [146], but the significance of these findings remains unclear.

Thiazolidinediones or Glitazones are a class of drugs commonly used to treat T2D by improving insulin sensitivity in peripheral tissues [147]. Several studies have investigated the impact of glitazone treatment on CaMKII in the heart. Glitazones (and/or curcumin) were shown to reduce the expression of CaMKII in diabetic cardiomyopathy in streptozotocin induced diabetic rats [148], but little evidence on human exist to support these findings.

EMPA-REG was an international, prospective, placebo-controlled clinical study investigating the cardiovascular outcome of empagliflozin, a sodium-glucose co-transporter-2 inhibitor (SGLT2i), in patients with heart failure and diabetes. This study showed that this treatment significantly reduced mortality [149], and similar results have been seen in diabetic and non-diabetic nephropathic patients [150, 151]. Thus, it is of considerable interest that SGLT2i appears to downregulate CaMKII activity [149, 152], and therefore myocyte CaMKII pathways may be a target for SGLT2i in diabetes [153].

Oxidation of CaMKII causing constitutive activation may be central to the toxic effects of mineralocorticoids e.g. aldosterone [154, 155]. Thus, steroidal mineralocorticoid receptor antagonists like spironolactone and eplerenone, and the non-steroidal mineralocorticoid antagonists (MRA), finerenone, may have some activity reducing CaMKII activity. The newer MRA have shown very good clinical results in patients with diabetes mellitus and CKD, especially more recently in the ARTS-DN/FIGARO-DKD/FIDELIO-DKD studies with improved renal and cardiac outcomes [156]. Whether or not this clinical improvement is due to MRA’s effects on the CaMKII pathways is not presently known.

The angiotensin receptor antagonist valsartan has been shown to be associated with inhibition of CaMKII in heart failure [157]. It is established that β-blockers are one of the standard therapeutic approaches for the treatment of chronic heart failure. While CaMKII activity is also increased by beta adrenergic stimulation (reviewed in [158]), β-blockers do not appear to downregulate CaMKII in experimental models [159].

Lipid pathways may also intersect with these signaling pathways and LDL appears to activate CaMKII [160], thus statins and other lipid lowering drugs might be expected to reduce CaMKII activation. The fact that we cannot show evidence for this suggests that this effect may be an epiphenomenon [161, 162].

Antioxidants in general might be expected to downregulate CaMKII activation, which makes it clinically important to counteract the CaMKII-induced upregulation of ROS [163]. Whilst many antioxidants have been shown to downregulate CaMKII pathways (e.g. resveratrol downregulates CaMKII in neurons), disappointingly untargeted antioxidant therapy has not been shown to therapeutically and materially affect outcomes, especially in diabetes [164]. On the other hand, studies have shown that intake of some phenolic compounds improves endothelial function and reduces CaMKII activity. A single ingestion of coffee polyphenols improved peripheral endothelial function after glucose loading in healthy subjects [165]. Furthermore, the 3′,4′-dihydroxyflavonol (DiOHF), a synthetic flavonol, was shown to alleviate diabetes-induced vascular dysfunction [166] and to modulate the p38 MAPK and JNK signaling cascades through inhibition of CaMKII with a potency (IC_50_ 0.25 μM) that is superior to that of the well-established CaMKII inhibitor, KN-93 (IC_50_ 3.3 μM) [167].

## Conclusion and future perspectives

**Fig. 2:**
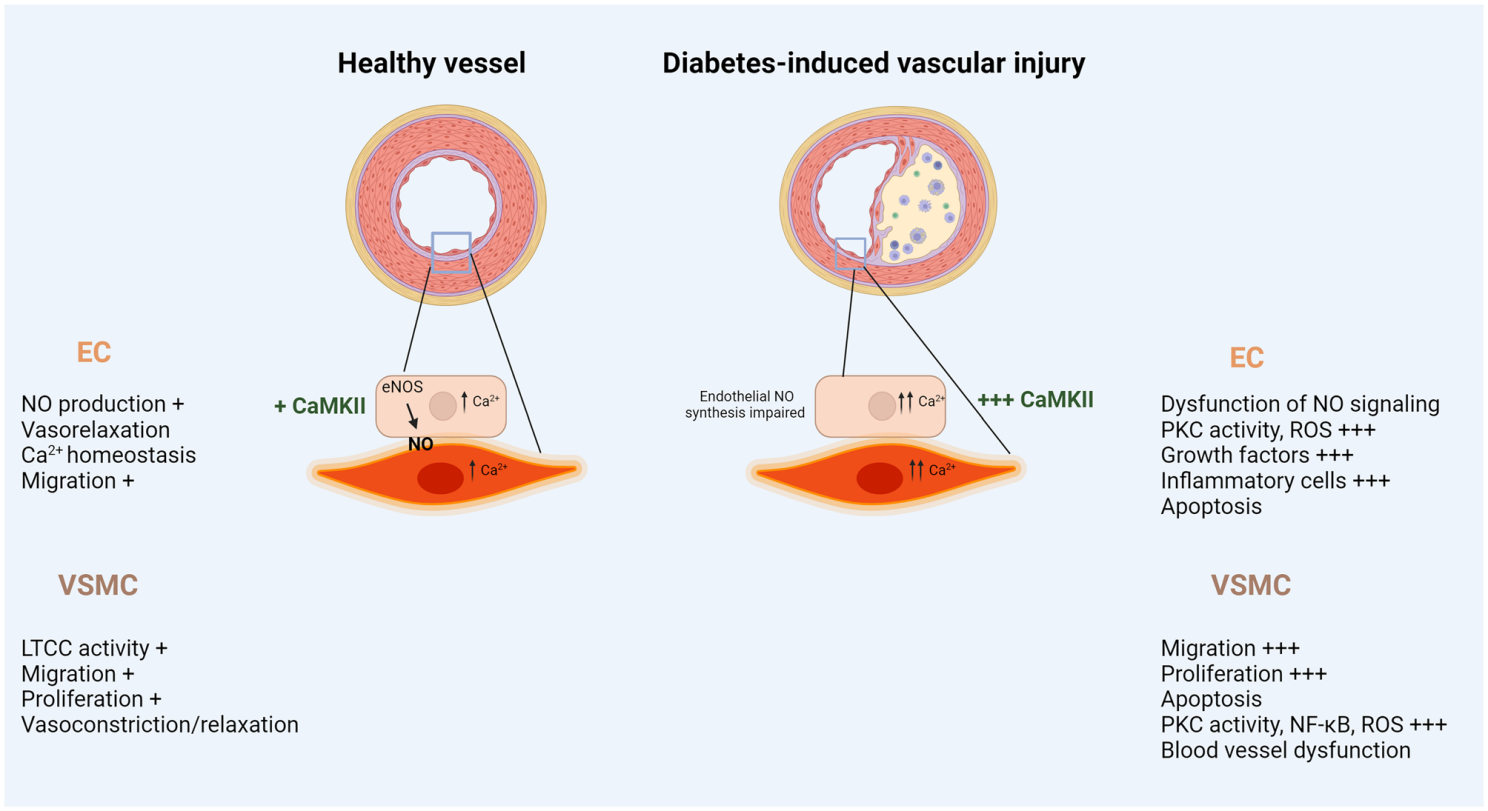
Role of CaMKII in healthy vessel and in diabetes-induced vascular injury. Abbreviations: CaMKII: Ca^2+^/calmodulin-dependent kinase II; eNOS: endothelial nitric oxide synthase; NO: nitric oxide; LTCC: L-type calcium channel; NF-κΒ: nuclear factor kappa B; PKC: protein kinase C; ROS: reactive oxygen species. Figure created with Biorender.com

There is a need to further characterize and define the role of CaMKII in regulating the interplay between ECs and VSMCs in diabetes. Chronic activation of CaMKII has been shown to be associated with inflammation, increased proliferation, migration and hypertrophy leading to abnormal vascular remodeling (Fig. 2). Whilst the current pharmacotherapy of diabetes is effective in abating hyperglycemia, the interaction of certain treatments with CaMKII should not be ignored. *In vivo* studies implicating animal models with targeted deletions of the CaMKII isoforms in the settings of diabetes are imperative to assess the changes in ECs and VSMCs under these conditions. These models could also serve as valuable tools to address the dynamics of EC/VSMC in the micro vasculatures (i.e. liver, retina, …etc) and the macrovasculature (i.e. coronary arteries). While targeting CaMKII signaling may offer a promising therapeutic approach for preventing or treating the observed complications in diabetes, delivering the different modalities of the discussed CaMKII inhibitors (drugs, siRNA, gene guided therapy-CRISPR Cas9, …etc) in a tissue-specific manner remains a major challenge. In fact, the advent of nanoparticles technology for drug delivery combined to genetic tools (i.e. siRNA or CRISPR Cas9) may provide a suitable solution to this complication. This opens the door for novel delivery methods of CaMKII inhibitors in patients with diabetes in a more effective and targeted manner. Nonetheless, the emergence of the mRNA-based vaccines may also reveal promising mostly by implicating the use of either RNAi for CaMKII isoforms, or expression cassettes for inhibitory peptides for CaMKII isoforms. In addition, a genetic screening of patients with diabetes for the various SNPs of the CaMKII isoforms may give hope for a better therapeutic approach to avoid vasculature-related complications. More research is needed to materialize these concepts, and a careful monitoring of the maladaptive remodeling of blood vessels is vitally important for patients with diabetes.

## Abbreviations

CaMKII: Ca^2+^/calmodulin-dependent kinase II
Calcium: Ca^2+^
ECs: Endothelial cells
VSMCs: Vascular smooth muscle cells
NO: Nitric oxide
PDGF: Platelet-derived growth factor
PKC: Protein kinase C
BH4: Tetrahydrobiopterin
eNOS: Endothelial nitric oxide synthase
iNOS: Inducible nitric oxide synthase
ROS: Reactive oxygen species
Cdk2: Cyclin-dependent kinase 2
CREB: cAMP responsive element binding protein
ERK: Extracellular-signal-regulated kinase
NF-κΒ: Nuclear factor kappa B
AMPK: Adenosine Monophosphate-Activated Protein Kinase
H_2_O_2_: hydrogen peroxide
IGF-1R: Insulin-like growth factor-1 receptor
TJ: Tight junctions
T2D: Type 2 diabetes
EPCs: Endothelial progenitor cells
MMP-9: Matrix metalloproteinase-9
miRNAs: MicroRNAs
SNPs: Single nucleotide polymorphisms
RNAi: RNA interference
CKD: Chronic kidney disease
MRA: Mineralocorticoid antagonists
SGLT2i: Sodium-glucose co-transporter-2 inhibitor
DiOHF: 3′,4′-dihydroxyflavonol
Epac: Exchange protein directly activated by cAMP
STOCs: Spontaneous transient outward currents
RyR: Ryanodine receptor
AIP: Autocamide-2-related inhibitory peptide
AC3-I: Autocamide-3 derived inhibitory peptide
